# pANCA autoantibody testing by indirect immunofluorescence indicates interstitial arteritis independent of MPO-ANCA immunoassays in ANCA-associated glomerulonephritis

**DOI:** 10.1007/s40620-022-01320-1

**Published:** 2022-04-05

**Authors:** Samy Hakroush, Ingmar Alexander Kluge, Eva Baier, Peter Korsten, Desiree Tampe, Philipp Ströbel, Björn Tampe

**Affiliations:** 1 Institute of Pathology, University Medical Center Göttingen, Göttingen, Germany; 2 Department of Nephrology and Rheumatology, University Medical Center Göttingen, Göttingen, Germany

**Keywords:** ANCA-associated glomerulonephritis, Indirect immunofluorescence, ANCA titer, Interstitial arteritis

The discovery of antineutrophil cytoplasmic antibodies (ANCAs) in ANCA-associated vasculitis (AAV) marked a breakthrough in diagnostics [1]. Subsequently, ANCAs were found in distinct small vessel vasculitides, including microscopic polyangiitis (MPA) and eosinophilic GPA (formerly known as Churg-Strauss syndrome). The current recommended diagnostic approach for ANCA testing includes immunoassays to detect myeloperoxidase (MPO)-ANCA or proteinase 3 (PR3)-ANCA used as the primary screening method for patients suspected of having AAV without the categorical need for indirect immunofluorescence (IIF). IIF occurs in three distinct patterns: cytoplasmic (cANCA), perinuclear (pANCA), and atypical (xANCA) [2]. A cANCA staining pattern is mainly linked to specificity for PR3, whereas a pANCA staining pattern for MPO [2]. ANCA IIF using ethanol-fixed unstimulated neutrophils as a substrate can also detect ANCA autoantibody binding to neutrophil autoantigens other than MPO or PR3, and these autoantibodies cannot be detected by specific MPO-ANCA or PR3-ANCA immunoassays [3]. We here aimed to directly compare validated ANCA testing by IIF, MPO-ANCA and PR3-ANCA immunoassays with histopathological findings in a cohort of 53 kidney biopsies with confirmed ANCA GN [4]. Detailed methods are described in Supplementary Materials and Methods.

In this cohort of biopsy-proven ANCA GN, 26/53 (49.1%) were MPO-ANCA GN and 27/53 (50.9%) were PR3-ANCA GN (Supplementary Table 1). In the subgroup of MPO-ANCA GN, pANCA IIF and respective MPO-ANCA titers measured by immunoassays correlated among each other (*p* < 0.0001, Supplementary Table 2). In contrast, a less robust association was observed for cANCA IIF and PR3-ANCA titers (*p* = 0.0899, Supplementary Table 2). Among other laboratory parameters, cANCA IIF correlated with C-reactive protein (CRP) elevation in PR3-ANCA GN (*p* = 0.0060, Fig. 1A, B and Supplementary Table 2). Beyond that, PR3-ANCA titers also correlated with complement system activation reflected by low serum levels of complement factor 4 (C4, *p* = 0.0107, Fig. 1B and Supplementary Table 2). We next aimed to describe the correlation between ANCA measurements and histopathological findings in ANCA GN (Fig. 1C). While pANCA IIF and MPO-ANCA measurements did not correlate with any glomerular, tubular or inflammatory lesions, specifically pANCA IIF correlated with interstitial arteritis (*v*) present in 7/26 (26.9%) of patients with MPO-ANCA GN (*p* = 0.0204, Fig. 1D, E and Supplementary Table 2). In PR3-ANCA GN, cANCA IIF and PR3-ANCA measurements were not associated with any glomerular, tubular or inflammatory lesions in this subgroup (Fig. 1F). In summary, ANCA autoantibody binding to neutrophil autoantigens as confirmed by pANCA IIF correlated specifically with interstitial arteritis regardless of the respective MPO-ANCA titers in a considerable subset of patients with ANCA GN.

**Fig. 1:**
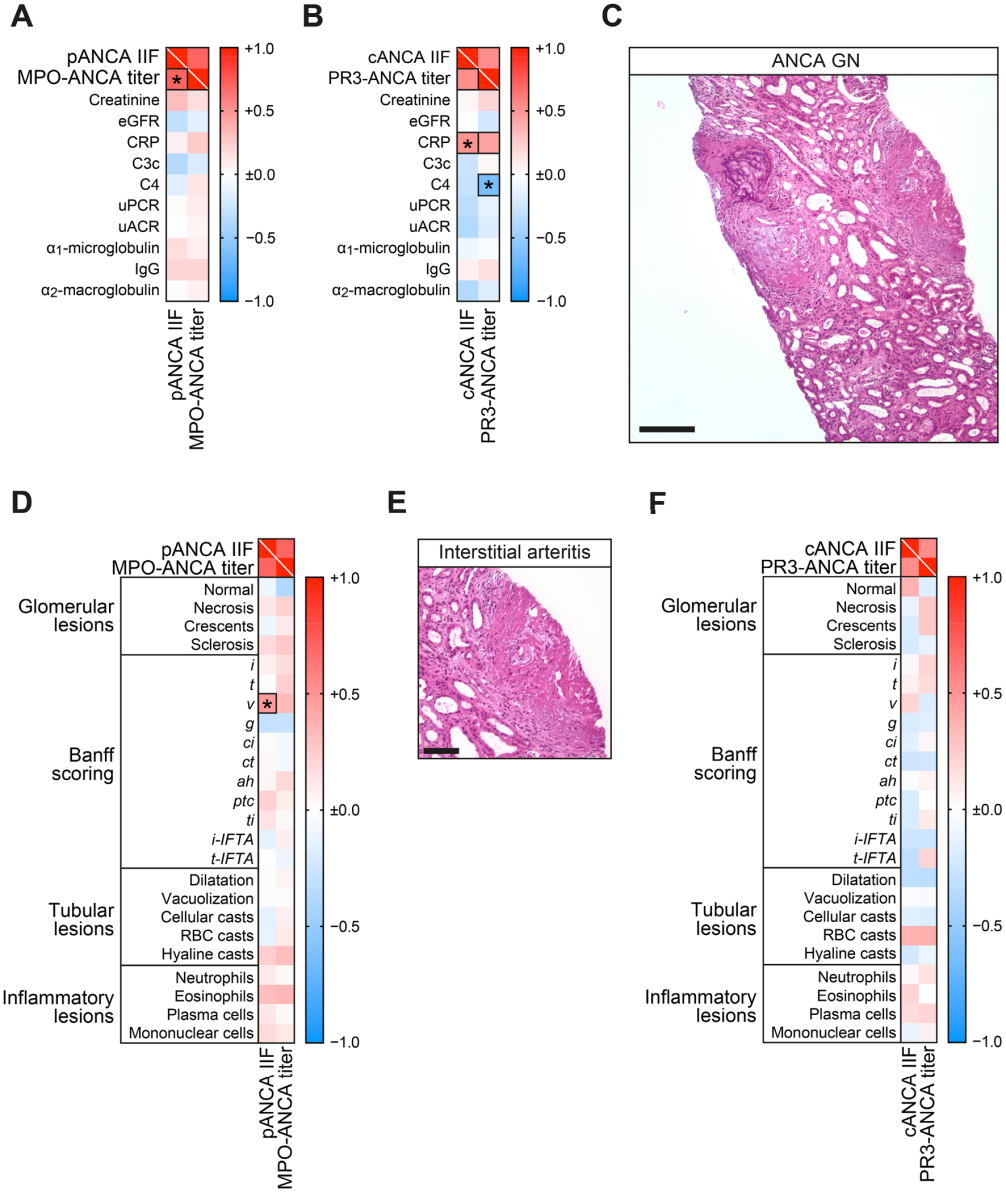
ANCA autoantibody testing by immunoassays and ANCA IIF associate with distinct clinical and laboratory parameters in ANCA GN. **A**, **B** Association between ANCA IIF and titers in association with laboratory parameters separated by MPO-ANCA GN and PR3-ANCA GN are shown by heatmap reflecting mean values of Spearman’s ρ. Rectangle boxes indicate a Spearman's ρ more than ± 0.4, asterisks indicate significant correlations in the linear regression analysis (*p* < *0.05*). **C** Representative kidney section stained for combined elastica and H&E in ANCA GN (scale bar: 200 µm), the inset shows interstitial arteritis in ANCA GN (scale bar: 100 µm). **D** Association between pANCA IIF and MPO-ANCA titers in association with histopathological findings are shown by heatmap reflecting mean values of Spearman’s ρ. The rectangle box indicates a Spearman's ρ more than ± 0.4, asterisk indicates a significant correlation in the linear regression analysis (*p* < *0.05*). **E** Representative kidney section stained for combined elastica and H&E showing interstitial arteritis in ANCA GN (scale bar: 100 µm). **F** Association between cANCA IIF and PR3-ANCA titers in association with histopathological findings are shown by heatmap reflecting mean values of Spearman’s ρ. *ah* arteriolar hyalinosis, *ANCA* anti-neutrophil cytoplasmic antibody, *BVAS* Birmingham Vasculitis Activity Score, *C3c* complement factor 3 conversion product, *C4* complement factor 4, *cANCA* cytoplasmatic ANCA, *ci* interstitial fibrosis, *CRP* C-reactive protein, *ct* tubular atrophy, *eGFR* estimated glomerular filtration rate (CKD-EPI), *g* glomerulitis, *GN* glomerulonephritis, *i* interstitial inflammation, *ICU* intensive care unit, *IgG* immunoglobulin G, *IIF* indirect immunofluorescence, *i-IFTA* inflammation in IFTA, *MPO* myeloperoxidase, *pANCA* perinuclear ANCA, *PR3* proteinase 3, *RBC* red blood cell, *SAPS II* Simplified Acute Physiology Score, *t* tubulitis, *ptc* peritubular capillaritis, *ti* total inflammation, *t-IFTA* tubulitis in IFTA, *uACR* urinary albumin-to-creatinine ratio, *uPCR* urinary protein-to-creatinine ratio, *v* arteritis

Besides ANCA testing for diagnostic value, its role as a disease activity marker has been the subject of frequent studies with varying outcomes. Discrepant results regarding disease activity have been a matter of discussion and can be interpreted by differences in ANCA epitopes and/or affinities [5]. On a mechanistic level, pathogenic ANCA autoantibodies can bind to the cell surface MPO of the proinflammatory cytokine-primed neutrophils, leading to excessive activation of neutrophils and subsequent destruction of the small vasculature [6]. Our finding that specifically pANCA IIF correlated with interstitial arteritis supports a pathomechanistic role of perinuclear targets, particularly MPO reflecting a neutrophil granule protein whose primary role in normal metabolic processes is the generation of oxygen radicals. It has previously been demonstrated that specifically MPO-ANCAs induce neutrophil extracellular traps (NETs) [6]. Autoantibodies against NET components trigger neutrophils to undergo NETosis, prompting tissue damage and autoimmunity in small vessel vasculitis including AAV [7]. The ability to induce NETs directly correlated with ANCA affinity to MPO and disease activity in ANCA GN [6]. Our observation that ANCA autoantibody binding to neutrophil autoantigens as confirmed by pANCA IIF regardless of the respective MPO-ANCA titers could imply that autoantigens other than MPO might contribute to neutrophil activation and a specific contribution for AAV manifestation to distinct renal compartments with interstitial arteritis in ANCA GN.

The main limitations of our study are its retrospective design, the small number of patients and lack of independent validation. Moreover, patients received steroids at the time of kidney biopsy that may have influenced the histopathological findings. Finally, quantification of additional ANCA autoantigens would further provide insights into a direct link between ANCA autoantibodies, neutrophil activation and AAV manifestation to distinct renal compartments (e.g., interstitial arteritis) in ANCA GN. Nevertheless, our finding that pANCA IIF specifically correlated with arteritis is especially relevant because arteritis determines renal prognosis in ANCA GN [8, 9]. Moreover, this unique association between pANCA IIF and specifically arteritis in MPO-ANCA GN regardless of glomerular or other tubulointerstitial lesions requires further investigation with regard to its pathomechanistic implications.

## Supplementary Information

Below is the link to the electronic supplementary material.

Supplementary file1 (PDF 103 KB)

## Data Availability

Deidentified data are available on reasonable request from the corresponding author.

## References

[1] Davies DJ (1982). Segmental necrotising glomerulonephritis with antineutrophil antibody: possible arbovirus aetiology?. Br Med J (Clin Res Ed).

[2] Bossuyt X (2017). Position paper: Revised 2017 international consensus on testing of ANCAs in granulomatosis with polyangiitis and microscopic polyangiitis. Nat Rev Rheumatol.

[3] Talor MV (2007). Antibodies to selected minor target antigens in patients with anti-neutrophil cytoplasmic antibodies (ANCA). Clin Exp Immunol.

[4] Hakroush S (2021). Comparative histological subtyping of immune cell infiltrates in MPO-ANCA and PR3-ANCA glomerulonephritis. Front Immunol.

[5] Suwanchote S (2018). Anti-neutrophil cytoplasmic antibodies and their clinical significance. Clin Rheumatol.

[6] Nakazawa D (2014). Enhanced formation and disordered regulation of NETs in myeloperoxidase-ANCA-associated microscopic polyangiitis. J Am Soc Nephrol.

[7] Kessenbrock K (2009). Netting neutrophils in autoimmune small-vessel vasculitis. Nat Med.

[8] Boudhabhay I (2021). Reappraisal of renal arteritis in ANCA-associated vasculitis: clinical characteristics, pathology, and outcome. J Am Soc Nephrol.

[9] Hakroush S, Tampe B (2021). Incidence of arteritis and peritubular capillaritis in ANCA-associated vasculitis. J Am Soc Nephrol.

