# Supplementary material for: pANCA autoantibody testing by indirect immunofluorescence indicates interstitial arteritis independent of MPO-ANCA immunoassays in ANCA-associated glomerulonephritis
Source: J Nephrol. 2022 Apr 5;35(4):1251–4. doi: 10.1007/s40620-022-01320-1 (PMC9107432; doi:10.1007/s40620-022-01320-1)
Supplement: Supplementary file 1 — Supplementary file1 (PDF 103 KB) [file 40620_2022_1320_MOESM1_ESM.pdf]

pANCA autoantibody testing by indirect immunofluorescence indicates interstitial arteritis independent of MPO-ANCA immunoassays in ANCA-associated glomerulonephritis

Supplementary Materials and Methods

Samy Hakroush[1], Ingmar Alexander Kluge[1], Eva Baier[2], Peter Korsten[2], Desiree Tampe[2], Philipp Ströbel[1], Björn Tampe[2*]

[1]*Institute of Pathology, University Medical Center Göttingen, Germany*

[2]*Department of Nephrology and Rheumatology, University Medical Center Göttingen, Germany*

Running title: pANCA IIF and interstitial arteritis

Corresponding author

Björn Tampe, MD

Department of Nephrology and Rheumatology

University Medical Center Göttingen

Robert Koch Street 40

37075 Göttingen, Germany

Email: bjoern.tampe@med.uni-goettingen.de

Supplementary Methods

Study population

A total number of 53 kidney biopsies with ANCA GN at the University Medical Center Göttingen were retrospectively included between 2015 till 2020, the patient cohort has previously been described [1-7]. All patients received only steroids at the time of kidney biopsy, further remission induction therapy was initiated thereafter based on clinical and histopathological presentation. The studies involving human participants were reviewed and approved by the Institutional Review Board of the University Medical Center Göttingen, Germany (no. 4/8/19). Informed written consent was obtained from all subjects involved in the study for the use of routinely collected data for research purposes as part of their regular medical care in the contract of the University Medical Center Göttingen.

Renal histopathology

Renal pathologists (SH and PS) separately evaluated all biopsies as were blinded to clinical data and analysis. Within a renal biopsy specimen, each glomerulus was scored separately for the presence of necrosis, crescents and global sclerosis. Consequently, the percentage of glomeruli with any of these features was calculated as a fraction of the total number of glomeruli in each renal biopsy. Renal biopsies were also evaluated analogous to the Banff scoring system for allograft pathology [8]. In brief, semi-quantitative Banff score lesions include interstitial inflammation (*i*), tubulitis (*t*), arteritis (*v*), glomerulitis (*g*), interstitial fibrosis (*ci*), tubular atrophy (*ct*), arteriolar hyalinosis (*ah*), peritubular capillaritis (*ptc*), total inflammation (*ti*), inflammation in areas of IFTA (*i-IFTA*) and tubulitis in areas of IFTA (*t-IFTA*) [8]. Systematic histological scoring of tubular injury lesions was evaluated as previously described [9, 10]. In brief, epithelial simplification and tubular dilation, non-isometric cell vacuolization, cellular, red blood cell (RBC), and hyaline casts were given a score ranging from 0 to 4 as a percentage of the total affected cortical area of the biopsy (score 0: <1%, 1: ≥1-10%, 2: ≥10-25%, 3: ≥25-

50%, 4: >50%). In addition, infiltrates of neutrophils, eosinophils, plasma cells, and mononucleated cells (macrophages, lymphocytes) were quantified as a fraction of the area of total cortical inflammation. The total cortical inflammation including areas of interstitial fibrosis and tubular atrophy, subcapsular and perivascular cortex including nodular infiltrates were considered.

ANCA IIF and autoantibody measurements

ANCA IIF was performed according to the manufacturer's protocol (EUROIMMUN AG, Lübeck, Germany), MPO-ANCA and PR3-ANCA autoantibodies were measured by immunoassay (ImmunoCAP 250, Thermo Fisher Scientific, Waltham, USA).

Statistical methods

Variables were tested for normal distribution using the Shapiro-Wilk test. Statistical comparisons were not formally powered or prespecified. Continuous and ordinal variables were presented as mean ± SD, categorical variables as percentages of total. For statistical analysis, reciprocal ANCA IIF titers were used. Spearman's correlation was performed to assess the correlation between ANCA autoantibody measurements and histopathological parameters, and heatmaps reflecting the mean values of Spearman's ρ are shown. A Spearman's ρ more than ±0.4 in the correlation matrix was defined as relevant indicated by rectangle boxes, and independent statistical evaluation of these parameters was performed. Data analyses were performed with GraphPad Prism (version 8.4.3 for MacOS, GraphPad Software, San Diego, California, USA). Stepwise multiple regression analyses were performed using IBM SPSS Statistics (version 27 for MacOS, IBM Corporation, Armonk, NY, USA). We retained covariates for ANCA IIF and titers with a Spearman's ρ more than ±0.4 in a linear regression model, limiting the model covariates to avoid model over-fit. A probability (p) value of <0.05 was considered statistically significant.

Supplementary References

1. Hakroush, S., et al., *Histopathological findings predict renal recovery in severe ANCA-associated vasculitis requiring intensive care treatment.* Front Med (Lausanne), 2020.
2. Hakroush, S., et al., *Systematic Histological Scoring Reveals More Prominent Interstitial Inflammation in Myeloperoxidase-ANCA Compared to Proteinase 3-ANCA Glomerulonephritis.* J Clin Med, 2021. **10**(6).
3. Tampe, D., et al., *Proteinuria Indicates Decreased Normal Glomeruli in ANCA-Associated Glomerulonephritis Independent of Systemic Disease Activity.* J Clin Med, 2021. **10**(7).
4. Hakroush, S., et al., *Bowman's capsule rupture links glomerular damage to tubulointerstitial inflammation in ANCA-associated glomerulonephritis.* Clin Exp Rheumatol, 2021.
5. Hakroush, S., et al., *Complement Components C3 and C4 Indicate Vasculitis Manifestations to Distinct Renal Compartments in ANCA-Associated Glomerulonephritis.* Int J Mol Sci, 2021. **22**(12).
6. Tampe, D., et al., *Comprehensive Analysis of Sex Differences at Disease Manifestation in ANCA-Associated Glomerulonephritis.* Front Immunol, 2021. **12**: p. 736638.
7. Hakroush, S., et al., *Comparative Histological Subtyping of Immune Cell Infiltrates in MPO-ANCA and PR3-ANCA Glomerulonephritis.* Front Immunol, 2021. **12**: p. 737708.
8. Roufosse, C., et al., *A 2018 Reference Guide to the Banff Classification of Renal Allograft Pathology.* Transplantation, 2018. **102**(11): p. 1795-1814.
9. Pieters, T.T., et al., *Histological characteristics of Acute Tubular Injury during Delayed Graft Function predict renal function after renal transplantation.* Physiol Rep, 2019. **7**(5): p. e14000.
10. Hakroush, S., et al., *Systematic Scoring of Tubular Injury Patterns Reveals Interplay between Distinct Tubular and Glomerular Lesions in ANCA-Associated Glomerulonephritis.* J Clin Med, 2021. **10**(12).

Supplementary Table 1. Clinical and histopathological parameters of the total ANCA GN cohort.

	Total cohort (n=53)	MPO-ANCA (n=26)	PR3-ANCA (n=27)
Clinical data			
Female sex – no. (%)	23 (43.4)	9 (34.6)	14 (51.9)
Age – years	62.4 ± 14.7	61 ± 13.3	63.7 ± 14.7
AAV relapse – no. (%)	8 (15.1)	5 (19.2)	3 (11.1)
BVAS – points	17.9 ± 4.2	17.2 ± 4.2	18.4 ± 4.2
Onset of disease – days prior admission	45.1 ± 78.8	55.9 ± 107.3	34.7 ± 33.6
ICU admission – no. (%)	24 (45.3)	12 (46.2)	12 (44.4)
Kidney injury			
Serum creatinine – mg/dL	3.4 ± 2.5	4.4 ± 2.8	2.5 ± 1.9
eGFR – mL/min	34.1 ± 32.3	24.5 ± 26.2	43.2 ± 35.3
RRT within 30 days after admission – no. (%)	16 (30.2)	9 (34.6)	7 (25.9)
ANCA class			
Crescentic class – no. (%)	17 (32.1)	10 (38.5)	7 (25.9)
Focal class – no. (%)	26 (49.1)	10 (38.5)	16 (59.3)
Mixed class – no. (%)	7 (13.2)	3 (11.5)	4 (14.8)
Sclerotic class – no. (%)	3 (5.7)	3 (11.5)	0 (0)
ARRS low risk – no. (%)	22 (41.5)	7 (26.9)	15 (55.6)
ARRS medium risk – no. (%)	23 (43.4)	12 (46.2)	11 (40.7)
ARRS high risk – no. (%)	8 (15.1)	7 (26.9)	1 (3.7)
Glomerular lesions			
Normal glomeruli – % of total	49.1 ± 28.8	39.1 ± 29.8	58.6 ± 24.7
Necrotic glomeruli – % of total	25.6 ± 28.3	27.7 ± 30.9	23.5 ± 26
Crescentic glomeruli – % of total	35.2 ± 28.6	40.8 ± 30.4	29.7 ± 26.1
Sclerotic glomeruli – % of total	16.8 ± 24.5	23.3 ± 31.6	10.6 ±12.6
Banff scoring			
i – Banff lesion score	0.18 ± 0.39	0.24 ± 0.44	0.13 ± 0.34
t – Banff lesion score	0.73 ± 0.84	0.84 ± 0.8	0.63 ± 0.88
v – Banff lesion score	0.5 ± 0.99	0.7 ± 1.08	0.32 ± 0.89
g – Banff lesion score	1.59 ± 0.93	1.44 ± 1.08	1.75 ± 0.74
ci – Banff lesion score	1.32 ± 0.87	1.62 ± 0.85	1.04 ± 0.81
ct – Banff lesion score	1.29 ± 0.71	1.56 ± 0.71	1 ± 0.59
ah – Banff lesion score	0.53 ± 0.83	0.63 ± 0.92	0.43 ± 0.73
ptc – Banff lesion score	0.1 ± 0.31	0.12 ± 0.33	0.08 ± 0.28
ti – Banff lesion score	0.86 ± 0.76	1.08 ± 0.64	0.63 ± 0.82
i-IFTA – Banff lesion score	1.84 ± 1.12	2 ± 1	1.67 ± 1.24
t-IFTA – Banff lesion score	0.69 ± 0.51	0.8 ± 0.5	0.58 ± 0.5
Tubular injury lesions			
Tubular dilatation – score	2.56 ± 1.32	2.92 ± 1.15	2.17 ± 1.4
Tubular vacuolization – score	0.19 ± 0.39	0.16 ± 0.37	0.22 ± 0.42
Tubular cellular casts – score	0.75 ± 0.79	0.84 ± 0.62	0.65 ± 0.93
Tubular RBC casts – score	0.52 ± 0.65	0.48 ± 0.65	0.57 ± 0.66
Hyaline casts – score	0.75 ± 0.76	0.92 ± 0.86	0.57 ± 0.59
Immune cell infiltration			
Neutrophils – % of total inflammation	0.87 ± 1.55	0.55 ± 0.96	1.17 ± 1.92
Eosinophils – % of total inflammation	0.53 ± 1.34	0.58 ± 1.59	0.47 ± 1.09
Plasma cells – % of total inflammation	3.1 ± 5.34	4.01 ± 6.7	2.26 ± 3.65
Mononuclear cells – % of total inflammation	10.1 ± 9.52	11.6 ± 8.67	8.78 ± 10.2

Abbreviations: AAV, ANCA-associated vasculitis; *ah*, arteriolar hyalinosis; ARRS, ANCA renal risk score; BVAS, Birmingham Vasculitis Activity Score; *ct*, tubular atrophy; *g*, glomerulitis; eGFR, estimated glomerular filtration rate (CKD-EPI); GN, glomerulonephritis; *i*, interstitial inflammation; ICU, intensive care unit; *i-IFTA*, inflammation in IFTA; MPO, myeloperoxidase; no., number; PR3, proteinase 3; RBC, red blood cell; RRT, renal replacement therapy; *t*, tubulitis; *ptc*, peritubular capillaritis; *ti*, total inflammation; *t-IFTA*, tubulitis in IFTA; *v*, intimal arteritis.

Supplementary Table 2. Linear regression analyses.

	ß	*SE*	*p value*
Comparison with ANCA IIF			
MPO-ANCA titers – IU/mL	0.8061	1.0021	*<0.0001*
PR3-ANCA titers – IU/mL	0.3327	1.3069	*0.0899*
Parameter associated with cANCA IIF			
CRP – mg/L	0.5144	0.6323	*0.0060*
Parameters associated with PR3-ANCA titers			
CRP – mg/L	-0.0083	0.1195	*0.9696*
C4 – g/L	-0.6394	115.6	*0.0107*
Parameter associated with cANCA IIF			
v – Banff lesion score	0.5141	69.9	*0.0204*

Abbreviations: ANCA, antineutrophil cytoplasmic antibody; C4, complement factor 4; cANCA, cytoplasmatic ANCA; CRP, C-reactive protein; MPO, myeloperoxidase; PR3, proteinase 3; SE, standard error; *v*, intimal arteritis.
